# Inter-organ crosstalk in systemic inflammation: a narrative review of molecular mechanisms and pharmacological modulation in the muscle–adipose–liver axis

**DOI:** 10.3389/fphar.2026.1849847

**Published:** 2026-09-14

**Authors:** Héctor Fuentes-Barría, Raúl Aguilera-Eguía, Miguel Alarcón-Rivera, Cherie Flores-Fernández, Lissé Angarita-Davila, Juan Alberto Aristizábal-Hoyos, Olga Patricia López-Soto

**Affiliations:** 1 Centro de Investigación en Medicina de Altura (CEIMA), Universidad Arturo Prat, Iquique, Chile; 2 Departamento de Salud Pública, Facultad de Medicina, Universidad Católica de la Santísima Concepción, Concepción, Chile; 3 Dirección de investigación, Universidad Bernardo O’Higgins, Santiago, Chile; 4 Departamento Gestión de la Información, Universidad Tecnológica Metropolitana, Santiago, Chile; 5 Escuela de Nutrición y Dietética, Facultad de Medicina, Universidad Andres Bello, Concepcion, Chile; 6 Departamento de Salud Oral, Facultad de Salud, Universidad Autónoma de Manizales, Manizales, Colombia

**Keywords:** cytokines, extracellular vesicles, metabolism, signal transduction, systemic inflammation

## Abstract

Systemic inflammation is increasingly recognized as a network-driven process sustained by dynamic inter-organ communication rather than isolated tissue responses. The muscle–adipose–liver axis plays a central role in this framework, integrating metabolic and immune signals that regulate systemic homeostasis. During inflammatory conditions, this coordinated communication becomes dysregulated, contributing to insulin resistance, chronic inflammation, and multi-organ dysfunction. Inter-organ crosstalk is mediated by multiple interconnected signaling layers, including cytokines, extracellular vesicles, lipid mediators, and metabolites, which collectively shape local and systemic responses. Emerging evidence highlights the importance of extracellular vesicles as long-distance carriers of molecular information, as well as the role of lipid mediators and metabolic reprogramming in linking inflammation with metabolic pathways. Pharmacological modulation of these networks represents a promising therapeutic strategy. While current interventions targeting cytokines and metabolic pathways show systemic benefits, newer approaches focusing on extracellular vesicle signaling and pro-resolving lipid mediators offer opportunities for more precise and context-dependent treatments. Advances in multi-omics and systems pharmacology are further enabling the identification of key regulatory nodes and biomarkers, supporting the development of precision medicine strategies. Overall, understanding and targeting inter-organ communication networks may provide more integrative and effective approaches for the treatment of systemic inflammatory and metabolic diseases.

## 1 Introduction

Systemic inflammation is increasingly recognized as a highly coordinated, multi-organ process driven by dynamic and reciprocal communication between tissues (Mou et al., 2022). Rather than being confined to a single anatomical site, inflammatory responses emerge from integrated signaling networks that connect peripheral organs with central metabolic and immune regulatory hubs (Bellocchi et al., 2022; Huang YW. et al., 2022). This concept of inter-organ crosstalk has reshaped our understanding of disease pathophysiology, highlighting that systemic outcomes—ranging from adaptive responses to multi-organ failure—are determined by the quality, intensity, and duration of these communication pathways (Huang YW. et al., 2022; Bain et al., 2023; Xie et al., 2024).

At the core of this network, skeletal muscle, adipose tissue, and the liver constitute a major axis of immune–metabolic regulation (Mai et al., 2024; Wu et al., 2025). These organs act as endocrine platforms that secrete a wide spectrum of signaling molecules, including cytokines, lipid mediators, metabolites, and extracellular vesicles (EVs), which collectively orchestrate local and systemic responses (Wu et al., 2025). Under physiological conditions, this coordinated signaling supports metabolic flexibility and immune homeostasis (Wu et al., 2025). However, during systemic inflammation—such as in obesity, type 2 diabetes, sepsis, and cancer-associated inflammation—this communication becomes dysregulated, contributing to chronic inflammation, insulin resistance, and progressive organ dysfunction (Fuentes-Barría et al., 2026; Paul et al., 2026; da et al., 2025).

Recent advances have expanded the classical view of soluble mediators by identifying extracellular vesicles as critical long-distance conveyors of biological information (Berumen et al., 2021). EVs transport proteins, lipids, and nucleic acids, enabling targeted intercellular communication and contributing to processes such as immune modulation, metabolic reprogramming, and organ-specific responses (Liu and Wang, 2023). In parallel, lipid mediators and eicosanoid signaling pathways, including cyclooxygenase (COX)- and lipoxygenase (LOX)-derived metabolites, have emerged as key integrators of local and systemic inflammation, linking cellular activation states with tissue-level outcomes (Chatzipieris et al., 2026; Wang et al., 2023; Ding et al., 2014). Additionally, metabolite-driven signaling has gained attention as a central mechanism in immunometabolic regulation, where shifts in metabolic pathways actively shape immune cell function and inter-organ communication, defined in this review as the exchange of molecular signals among anatomically distinct organs that coordinate systemic metabolic, immune, and physiological responses (Chi, 2022; Xu R. et al., 2024).

Importantly, these signaling modalities do not operate independently but are embedded within complex, multilayered networks characterized by feedback loops, organotropism, and context-dependent responses (Xu R. et al., 2024; Benito-Lopez et al., 2023; Garrido-Rodriguez et al., 2022). The integration of multi-omics approaches, including transcriptomics, metabolomics, lipidomics, and EV profiling, has enabled the identification of novel biomarkers and mechanistic nodes that govern inter-organ communication (Shaba et al., 2022; Meng et al., 2024; Liu Y. et al., 2023). These advances provide new opportunities to better understand the transition from adaptive to maladaptive inflammation and to identify targets for therapeutic intervention (Li et al., 2026).

From a pharmacological perspective, modulating inter-organ crosstalk, defined in this review as the dynamic and often bidirectional functional interactions among organs that arise from inter-organ communication, represents a promising yet still underexplored strategy (Zhang et al., 2026). Beyond traditional anti-inflammatory therapies, emerging interventions aim to target specific signaling axes, including EV-mediated communication, receptor-selective modulation of lipid mediators, and metabolic pathway reprogramming (Zhang et al., 2026; Mimi and Hasan, 2025; Kolawole and Kashfi, 2025). Drugs such as metformin, GLP-1 receptor agonists, and SGLT2 inhibitors exemplify how systemic metabolic and inflammatory pathways can be simultaneously influenced, although their effects on inter-organ communication networks remain incompletely characterized (González-Casanova et al., 2025; Ngabea and Dimeji, 2025; Mashayekhi et al., 2024).

In this narrative review, we provide an integrative overview of the molecular mechanisms underlying inter-organ crosstalk during systemic inflammation, with a particular focus on the muscle–adipose–liver axis. We discuss the roles of extracellular vesicles, lipid mediators, and metabolites as key drivers of systemic communication, and highlight emerging pharmacological strategies aimed at modulating these interconnected networks. Finally, we address current challenges and future directions, emphasizing the potential of systems-level approaches to identify novel therapeutic targets and improve clinical outcomes in inflammatory and metabolic diseases.

## 2 Molecular mediators of Inter-organ crosstalk during systemic inflammation

### 2.1 Cytokines and classical inflammatory signaling

Cytokines represent the canonical layer of inter-organ communication during systemic inflammation, acting as rapidly inducible, pleiotropic mediators that coordinate immune, metabolic, and vascular responses across tissues (Chauhan et al., 2021). Their systemic relevance lies not only in their circulating concentrations but also in their spatiotemporal dynamics, receptor distribution, and downstream signaling specificity. Among these, interleukin-6 (IL-6), tumor necrosis factor-α (TNF-α), and interleukin-1β (IL-1β) constitute a core triad that integrates innate immune activation with metabolic regulation (de Baat et al., 2023). Although these cytokines are classically secreted by activated immune cells, including macrophages, monocytes, neutrophils, and T lymphocytes, they are also produced by adipocytes, skeletal muscle fibers, hepatocytes, Kupffer cells, hepatic stellate cells, and other resident stromal cells in response to metabolic or inflammatory stress. The relative contribution of immune-cell-derived *versus* tissue-derived cytokines varies according to disease stage, nutritional status, and the local tissue microenvironment, thereby influencing the magnitude and direction of inter-organ inflammatory signaling (Wang Y. et al., 2026; Cai et al., 2026).

At the molecular level, cytokine signaling is mediated through well-defined pathways such as JAK/STAT, NF-κB, and MAPK cascades, which regulate transcriptional programs associated with inflammation, cellular stress, and metabolic adaptation (Xin et al., 2020; Nagoor Meeran et al., 2023). IL-6 signaling exemplifies the complexity of cytokine action through its dual modes: classical signaling *via* membrane-bound IL-6 receptor (IL-6R) and trans-signaling *via* soluble IL-6R, the latter being strongly associated with pro-inflammatory systemic effects (Uciechowski and Dempke, 2020). This distinction is critical, as muscle-derived IL-6 released during exercise primarily engages metabolic and anti-inflammatory pathways, whereas chronic IL-6 elevation in obesity and sepsis promotes hepatic acute-phase responses, insulin resistance, and endothelial dysfunction (Yang et al., 2025). Within the muscle-adipose-liver axis, this functional dichotomy illustrates how the tissue of origin determines the physiological consequences of cytokine signaling (Lin et al., 2023). Skeletal muscle-derived IL-6 acts as a myokine that promotes hepatic glucose output during exercise while enhancing lipid oxidation and exerting anti-inflammatory effects in adipose tissue. In contrast, IL-6 released from inflamed adipose tissue contributes to hepatic insulin resistance and chronic low-grade systemic inflammation (Lin et al., 2023; Leal et al., 2018).

TNF-α and IL-1β act as potent amplifiers of inflammatory cascades. TNF-α disrupts insulin signaling through serine phosphorylation of IRS-1 and activation of stress kinases such as JNK, thereby linking inflammation to metabolic dysfunction (Craig et al., 2025). IL-1β, generated through inflammasome activation (particularly NLRP3), drives local and systemic inflammation by inducing secondary cytokine production and endothelial activation (Paik et al., 2021). Adipose tissue represents a major source of TNF-α and IL-1β during obesity, where cytokine release by hypertrophic adipocytes and infiltrating macrophages impairs hepatic insulin signaling, stimulates gluconeogenesis and lipid accumulation, and reduces glucose uptake in skeletal muscle. Conversely, cytokines and myokines released from contracting skeletal muscle influence adipose tissue lipolysis and hepatic substrate utilization, whereas liver-derived inflammatory mediators and hepatokines can feed back to skeletal muscle and adipose tissue, further modulating systemic metabolic homeostasis (Jensen-Cody and Potthoff, 2021; de Oliveira Dos Santos et al., 2021). Importantly, these cytokines contribute to inter-organ propagation of inflammation, whereby signals originating in adipose tissue or immune cells influence hepatic glucose production, skeletal muscle metabolism, and vascular function (Vélez et al., 2023).

Cytokine networks are further shaped by feedback and feedforward loops that determine the transition from adaptive to maladaptive responses (Saha et al., 2023). For instance, persistent TNF-α signaling sustains NF-κB activation, promoting chronic inflammation, while IL-6 trans-signaling reinforces leukocyte recruitment and tissue damage (Kalliolias and Ivashkiv, 2016). Anti-inflammatory cytokines such as IL-10 and TGF-β counterbalance these effects but are often insufficient in chronic disease states (Bobhate et al., 2021).

From a systems perspective, cytokines function as network hubs that integrate signals from extracellular vesicles, metabolites, and lipid mediators, reinforcing the concept that classical inflammatory signaling is embedded within a broader inter-organ communication framework (Medzhitov, 2021). Rather than acting as isolated mediators, cytokines orchestrate bidirectional communication among skeletal muscle, adipose tissue, and the liver, coordinating metabolic adaptation under physiological conditions while propagating systemic inflammation when signaling becomes chronic or dysregulated (de Oliveira Dos Santos et al., 2021). Therapeutically, targeting cytokine pathways has shown clinical efficacy; however, the challenge remains to selectively modulate pathological signaling while preserving physiological and adaptive functions (Leonard and Lin, 2023). This highlights the need for context-dependent and pathway-selective interventions, particularly those distinguishing between protective and deleterious cytokine signaling modes (Leonard and Lin, 2023; Wiedemann et al., 2021).

### 2.2 Extracellular vesicles as long-distance messengers

EVs have emerged as a fundamental mechanism of inter-organ communication, enabling the transfer of complex molecular information beyond the constraints of soluble factor diffusion (Zhang Y. et al., 2024). EVs encompass heterogeneous populations, primarily exosomes (30–150 nm) and microvesicles (100–1,000 nm), which differ in biogenesis but share the capacity to transport proteins, lipids, metabolites, and nucleic acids, including regulatory microRNAs and long non-coding RNAs (Lange, 2023).

The biogenesis of EVs is tightly regulated and highly responsive to inflammatory stimuli (Das et al., 2023). Exosomes originate from the endosomal pathway through multivesicular body formation, involving endosomal sorting complex required for transport (ESCRT) dependent and ESCRT independent mechanisms, while microvesicles are generated *via* direct plasma membrane budding (Bavafa et al., 2025). During systemic inflammation, extracellular vesicle biogenesis and cargo sorting are regulated by the ESCRT machinery and intracellular signaling pathways, including NF-κB and hypoxia-inducible factors, resulting in quantitative and qualitative changes in EV release and cargo composition (Zhang J. et al., 2022; Li et al., 2021).

A defining feature of EV-mediated communication is cargo selectivity, which is not random but actively regulated. For example, specific microRNAs associated with inflammation (e.g., miR-155, miR-21) are preferentially enriched in EVs under inflammatory conditions (Park et al., 2016). For instance, adipose tissue-derived EVs enriched in miR-155 are released into the circulation and taken up by skeletal muscle, where they impair insulin signaling through suppression of PPARγ and downstream insulin-responsive pathways (Ji et al., 2025). Similarly, hepatocyte-derived EVs carrying miR-122 and inflammatory proteins are internalized by macrophages and adipose tissue, promoting inflammatory activation and disrupting systemic lipid metabolism (Huang and Xu, 2021). In contrast, skeletal muscle-derived EVs released during exercise transport regulatory microRNAs, proteins, and metabolites to the liver and adipose tissue, where they enhance fatty acid oxidation, improve insulin sensitivity, and attenuate inflammatory signaling (Ji et al., 2025; Huang and Xu, 2021). Adipose tissue-derived EVs carrying such cargo can impair insulin signaling in skeletal muscle by targeting key metabolic regulators, while liver-derived EVs can modulate immune cell activation and systemic lipid metabolism (Stevanović et al., 2025). Conversely, EVs released during exercise contain anti-inflammatory and metabolic regulatory signals that contribute to improved inter-organ communication (Verboven and Vechetti, 2023). Most mechanistic evidence supporting these pathways has been obtained from experimental animal models and *in vitro* studies, whereas human studies have largely confirmed the presence and differential cargo of circulating EVs but remain more limited in establishing causal inter-organ signaling mechanisms (Mathieu et al., 2019; Kalluri and LeBleu, 2020).

EVs also exhibit organotropism, mediated by surface molecules such as integrins and tetraspanins that determine their uptake by specific tissues. This property is particularly relevant in pathological contexts such as cancer, where tumor-derived EVs contribute to pre-metastatic niche formation by reprogramming distant tissues, modulating immune responses, and altering vascular permeability (Garofalo et al., 2021; Kalluri, 2024; Dong et al., 2024).

From a translational perspective, EVs represent both biomarkers and therapeutic tools (Urabe et al., 2020; Louie et al., 2023). Their molecular cargo reflects the physiological or pathological state of the tissue of origin, enabling early detection of organ dysfunction (de Miguel-Perez et al., 2024). Moreover, engineered EVs are being explored as delivery systems for drugs, RNA-based therapeutics, and gene-editing tools, offering high specificity and reduced immunogenicity (Muskan et al., 2024).

Despite these advances, challenges remain in standardizing EV isolation, characterization, and functional analysis (Veerman et al., 2021). Importantly, EV-mediated communication is tightly interconnected with other signaling layers discussed throughout this review. Pro-inflammatory cytokines such as TNF-α and IL-6 regulate EV biogenesis and cargo composition, whereas EVs transport cytokines, lipid mediators, metabolic enzymes, and regulatory microRNAs that amplify or attenuate inflammatory responses in recipient tissues (Dixson et al., 2023; Jin et al., 2022). Likewise, metabolic reprogramming induced by obesity, exercise, or insulin resistance modifies both EV release and molecular, thereby coordinating communication between skeletal muscle, adipose tissue, and the liver (Snyder et al., 2021; Deng et al., 2009). These observations indicate that extracellular vesicles should not be considered isolated signaling entities but rather integral components of an interconnected communication network that links inflammatory, metabolic, and endocrine signaling across organs. A deeper understanding of EV biology, particularly in the context of systemic inflammation, is essential to fully exploit their potential as modulators of inter-organ crosstalk and as targets for pharmacological intervention (Pan et al., 2022).

### 2.3 Lipid mediators and eicosanoid networks

Lipid mediators derived from polyunsaturated fatty acids, particularly arachidonic acid, constitute a highly dynamic signaling system that integrates local inflammatory responses with systemic physiological outcomes (Brennan et al., 2021). The enzymatic conversion of arachidonic acid through COX, LOX, and cytochrome P450 pathways generates a diverse array of bioactive lipids, including prostaglandins, thromboxanes, leukotrienes, and specialized pro-resolving mediators (SPMs) such as resolvins, protectins, and maresins (Kolawole and Kashfi, 2025).

These lipid mediators exert their effects through G protein-coupled receptors (GPCRs) and nuclear receptors, enabling rapid and context-dependent modulation of cellular function (Patwardhan et al., 2021). Prostaglandins, for instance, regulate vascular tone, fever response, and immune cell activation, while leukotrienes are potent mediators of leukocyte recruitment and vascular permeability (Horikami et al., 2020). In contrast, SPMs actively promote the resolution of inflammation by enhancing macrophage-mediated clearance, reducing neutrophil infiltration, and restoring tissue homeostasis (Homer et al., 2025).

A key feature of eicosanoid signaling is its temporal organization, often described as a “class switch” from pro-inflammatory to pro-resolving mediators (Serhan and Levy, 2018). Disruption of this switch is a hallmark of chronic inflammatory diseases, where persistent production of pro-inflammatory lipid mediators coexists with impaired resolution pathways (Homer et al., 2025; Serhan and Levy, 2018). This imbalance contributes to sustained inter-organ signaling dysfunction, particularly in metabolic diseases such as obesity and type 2 diabetes (de Gaetano et al., 2018).

Lipid mediators also participate in inter-organ communication by modulating endocrine and paracrine signaling networks (Regidor et al., 2020; Chatterjee et al., 2017). For instance, during obesity, hypertrophic adipocytes and adipose tissue macrophages increase the production of pro-inflammatory eicosanoids, including prostaglandin E2 (PGE2) and leukotriene B4 (LTB4), which enter the circulation and promote hepatic inflammation, impair insulin signaling, and reduce glucose uptake in skeletal muscle. Conversely, the liver produces lipid mediators such as specialized pro-resolving mediators (SPMs), including resolvins and lipoxins, which can act on adipose tissue and immune cells to attenuate inflammatory responses and promote the resolution of tissue inflammation (Chen et al., 2025). Furthermore, circulating free fatty acids released from adipose tissue activate Toll-like receptor 4 (TLR4) signaling in hepatocytes and skeletal muscle, further linking lipid metabolism with inflammatory signaling across organs. Interactions between lipid mediators and cytokine signaling pathways create complex regulatory circuits that amplify or dampen inflammatory responses (Mayer-Barber and Sher, 2015).

Pharmacologically, targeting eicosanoid pathways has long been a cornerstone of anti-inflammatory therapy. Non-steroidal anti-inflammatory drugs (NSAIDs) inhibit COX enzymes, reducing prostaglandin synthesis, whereas leukotriene receptor antagonists target LOX-derived pathways (Wirth et al., 2024; P et al., 2018). More recently, therapeutic strategies have shifted toward enhancing pro-resolving pathways, including the use of SPM analogs and receptor agonists, aiming to restore physiological resolution rather than simply suppress inflammation (Basil and Levy, 2016).

Collectively, lipid mediators do not function as isolated signaling molecules but rather as integral components of a coordinated inter-organ communication network. Cytokine signaling can reshape eicosanoid biosynthesis by regulating the expression of COX and LOX enzymes, whereas extracellular vesicles released from skeletal muscle, adipose tissue, and the liver transport lipid mediators, lipid-metabolizing enzymes, and regulatory microRNAs that further modulate lipid signaling in recipient organs (Thulasingam and Haeggström, 2020; Izzotti et al., 2020). Conversely, metabolic reprogramming associated with obesity, insulin resistance, or exercise alters both lipid mediator production and extracellular vesicle, thereby coordinating inflammatory and metabolic responses across the muscle–adipose–liver axis (Lee and Olefsky, 2021). This reciprocal integration highlights that lipid mediators, cytokines, extracellular vesicles, and metabolites operate as interconnected signaling layers rather than independent pathways, collectively orchestrating systemic inflammatory and metabolic homeostasis.

Understanding the balance between pro-inflammatory and pro-resolving lipid mediators is therefore critical for developing more effective and selective interventions that modulate inter-organ crosstalk without compromising host defense mechanisms (Serhan, 2014).

### 2.4 Metabolite-driven immunometabolic communication

Metabolites are increasingly recognized as central regulators of immune function and inter-organ communication, bridging cellular metabolism with systemic inflammatory responses (Rukonge et al., 2026). During systemic inflammation, profound metabolic reprogramming occurs across multiple tissues, leading to alterations in the production, release, and utilization of key metabolites such as glucose, lactate, free fatty acids, ketone bodies, and amino acid derivatives (Liu W. et al., 2023).

This metabolic shift is closely linked to the functional state of immune cells. Activated immune cells adopt distinct metabolic profiles—such as aerobic glycolysis in pro-inflammatory macrophages—resulting in the accumulation of metabolites that act as signaling molecules (Wang et al., 2018). Lactate, for example, modulates immune cell polarization, suppresses cytotoxic T cell activity, and influences cytokine production, thereby contributing to the regulation of inflammatory responses beyond its traditional role as a metabolic byproduct (Fang et al., 2024).

Similarly, lipid-derived metabolites, including ceramides and diacylglycerols, play critical roles in the development of insulin resistance by interfering with intracellular signaling pathways in skeletal muscle and liver (Ibrahim et al., 2017). Branched-chain amino acids (BCAAs) and their metabolites have also been implicated in metabolic inflammation, linking nutrient sensing to immune activation and inter-organ communication (Melnik, 2015).

The concept of immunometabolism emphasizes the bidirectional interaction between metabolic and immune pathways (Shi et al., 2024). Organs such as the liver, skeletal muscle, and adipose tissue function as both sources and targets of metabolite signaling, forming a dynamic network that adapts to physiological and pathological conditions. For instance, hepatic gluconeogenesis, adipose tissue lipolysis, and muscle glucose uptake are tightly regulated by systemic metabolic cues that are, in turn, influenced by inflammatory signals (He et al., 2021).

At a systems level, metabolite-driven communication integrates with cytokine, EV, and lipid mediator networks, forming a multilayered regulatory architecture (Boilard, 2018). Advances in metabolomics and flux analysis have enabled the identification of key metabolic nodes that govern these interactions, providing new opportunities for therapeutic intervention (L et al., 2021).

Pharmacologically, targeting metabolic pathways offers a promising strategy to modulate systemic inflammation (Arthur and Ley, 2013). Agents such as metformin influence mitochondrial function and AMP-activated protein kinase (AMPK) signaling, while newer therapies aim to directly modulate metabolic intermediates or their signaling pathways (Jung et al., 2018). These approaches highlight the potential of metabolite-centered interventions to reshape inter-organ crosstalk and restore homeostasis in inflammatory and metabolic diseases (Carling, 2017).

Building upon the central role of metabolic pathways in modulating systemic inflammation, inter-organ crosstalk emerges as a highly integrated network driven by multiple layers of molecular signaling. These interconnected pathways involve cytokines, extracellular vesicles, lipid mediators, and metabolites, each contributing distinct yet complementary roles in coordinating systemic responses across tissues. While individual mediators have been extensively studied, their combined effects within a network framework are critical for understanding the transition from physiological regulation to pathological dysregulation. In this context, Table 1 summarizes the key molecular mediators involved in inter-organ communication, highlighting their sources, targets, and principal biological effects.

**TABLE 1:** Key molecular mediators of inter-organ crosstalk in systemic inflammation.

Mediator type	Key molecules	Source organs	Target organs	Main effects
Cytokines	IL-6, TNF-α, IL-1β	Muscle, adipose tissue, immune cells	Liver, muscle, vasculature	Inflammation and insulin resistance
Extracellular vesicles	miR-155, miR-21, proteins	Adipose tissue, liver, muscle	Muscle, liver, immune cells	Signal transfer and metabolic regulation
Lipid mediators	Prostaglandins, leukotrienes, SPMs	Immune cells, adipose tissue	Multiple organs	Inflammation and resolution
Metabolites	Lactate, FFAs, BCAAs	Muscle, liver, adipose tissue	Immune cells, liver, muscle	Immunometabolic regulation

Source: own elaboration.

Complementarily, Figure 1 provides an integrated schematic representation of these signaling layers, illustrating how they converge to orchestrate systemic inflammation through dynamic and reciprocal interactions between organs.

**FIGURE 1:**
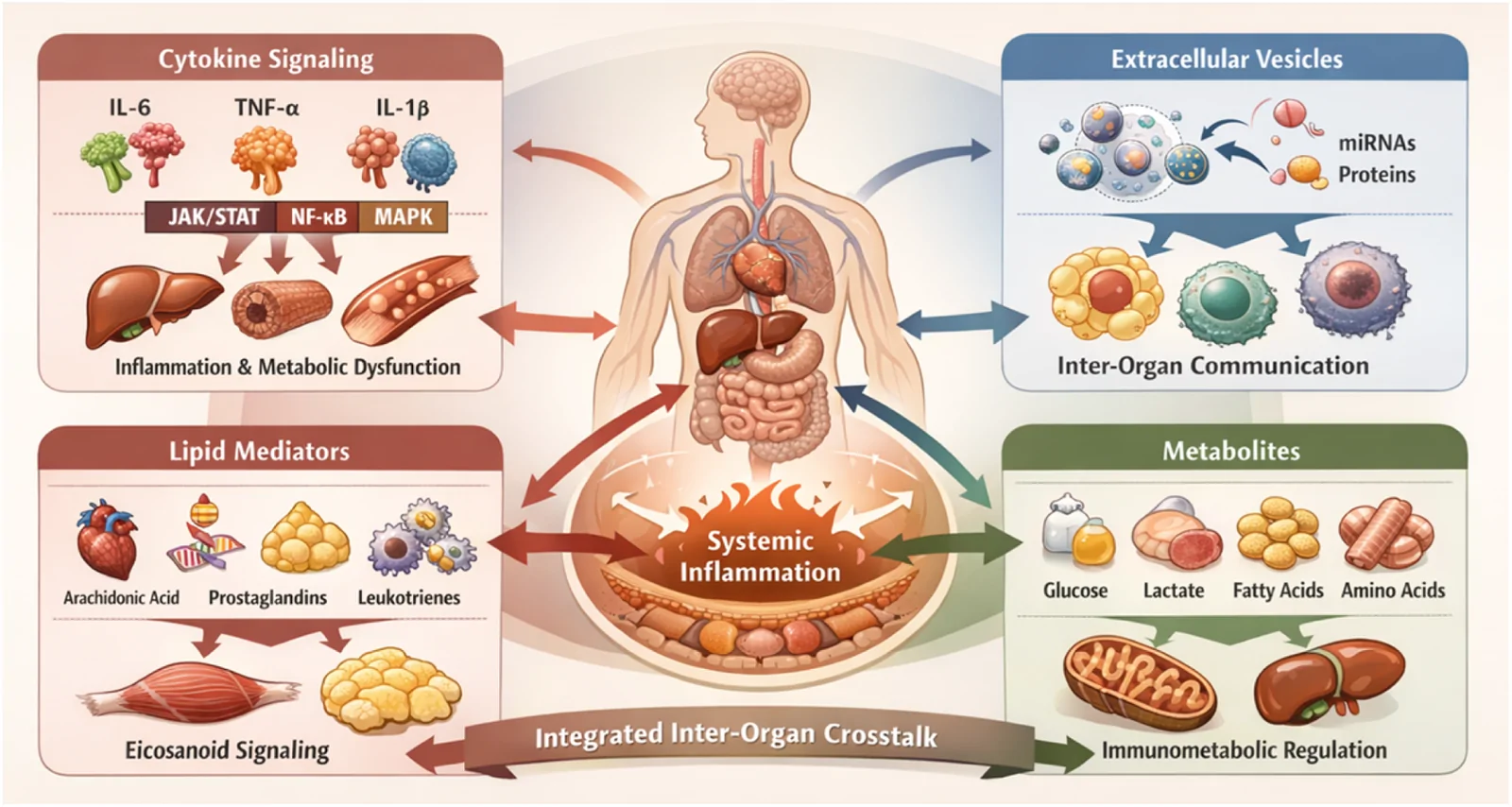
Molecular mediators of inter-organ crosstalk during systemic inflammation. Source: own elaboration.

## 3 The muscle–adipose–liver axis in systemic inflammation

The skeletal muscle–adipose tissue–liver axis constitutes a central regulatory network that integrates metabolic and inflammatory signals during both physiological adaptation and pathological states (Li A. et al., 2025). These organs engage in continuous bidirectional communication through endocrine, paracrine, and vesicle-mediated mechanisms, coordinating energy homeostasis, immune responses, and substrate utilization (Yue et al., 2022). During systemic inflammation, this tightly regulated network becomes profoundly dysregulated, leading to metabolic inflexibility, chronic low-grade inflammation, and progressive organ dysfunction (Li, 2023).

A defining feature of this axis is its functional plasticity, allowing rapid adaptation to environmental and metabolic stressors. However, persistent inflammatory stimuli—such as those observed in obesity, sepsis, and cancer-associated cachexia—shift this system toward maladaptive signaling, characterized by altered secretion of myokines, adipokines, and hepatokines, as well as disrupted metabolic fluxes (Smith et al., 2018). Importantly, these alterations are not isolated but propagate across organs, amplifying systemic dysfunction through interconnected feedback loops (Le Lay and Scherer, 2025).

### 3.1 Skeletal muscle as an endocrine organ

Skeletal muscle is increasingly recognized as a dynamic endocrine organ capable of modulating systemic physiology through the secretion of myokines (Hah et al., 2026). These signaling molecules are released in response to mechanical, metabolic, and inflammatory stimuli, enabling skeletal muscle to communicate with distant organs such as adipose tissue, liver, pancreas, and the immune system (Hah et al., 2026).

At the molecular level, myokine secretion is regulated by pathways including AMPK, PGC-1α, and NF-κB, which serve as upstream regulators of distinct myokine programs rather than uniformly controlling the expression of a single myokine. AMPK and PGC-1α are primarily activated during exercise and coordinate metabolic adaptation, mitochondrial biogenesis, and oxidative metabolism, whereas NF-κB is predominantly activated by inflammatory and cellular stress signals, promoting the expression of pro-inflammatory myokines. Consequently, activation of these pathways generates different myokine profiles depending on the physiological or pathological context (Zhang Y. et al., 2022). Within this regulatory framework, IL-6 remains the prototypical myokine, exhibiting context-dependent effects. During acute exercise, AMPK- and PGC-1α-associated signaling promotes the transient release of muscle-derived IL-6, which enhances glucose uptake, fatty acid oxidation, and anti-inflammatory signaling, partly through the induction of IL-10 and inhibition of TNF-α (Pinto et al., 2022; Calegari et al., 2018). In contrast, chronic elevation of IL-6 in inflammatory states, largely associated with sustained NF-κB activation, contributes to insulin resistance and hepatic gluconeogenesis, underscoring the importance of temporal dynamics in myokine signaling (Barrett, 2024).

Other myokines such as irisin and myostatin further illustrate the complexity of muscle-derived endocrine signaling, which in this review refers to the secretion of bioactive molecules into the circulation that act on distant target organs, thereby mediating inter-organ communication through endocrine mechanisms. Irisin, produced through cleavage of FNDC5, promotes browning of white adipose tissue and enhances energy expenditure, linking muscle activity to systemic metabolic regulation (Waseem et al., 2022). Myostatin, a negative regulator of muscle growth, is upregulated in catabolic states and contributes to muscle wasting, insulin resistance, and altered lipid metabolism (Yang et al., 2023).

During systemic inflammation, skeletal muscle undergoes profound metabolic and structural alterations, including proteolysis, mitochondrial dysfunction, and reduced anabolic signaling, which collectively contribute to sarcopenia and cachexia (Zhang HJ. et al., 2024). These changes are accompanied by a shift in myokine profiles toward a pro-inflammatory phenotype, impairing inter-organ communication and reinforcing systemic metabolic dysfunction (Iglesias, 2025).

Importantly, skeletal muscle-derived signals interact with immune cells, influencing macrophage polarization and T cell responses, thereby positioning muscle as a key regulator of immunometabolic homeostasis (Wu et al., 2020). From a therapeutic perspective, interventions such as exercise and exercise-mimetic drugs have the potential to restore beneficial myokine signaling, representing a promising strategy to modulate inter-organ crosstalk in inflammatory diseases (Samant and Prabhu, 2024).

### 3.2 Adipose tissue as an immunometabolic hub

Adipose tissue is a highly active endocrine and immune organ that plays a central role in systemic inflammation and metabolic regulation (Xu S. et al., 2024). Beyond its function in energy storage, adipose tissue integrates nutrient sensing, hormonal signaling, and immune responses, acting as a critical hub in inter-organ communication (Takahashi et al., 2024).

Adipocytes secrete a diverse array of adipokines, including leptin, adiponectin, resistin, and visfatin, which exert systemic effects on appetite regulation, insulin sensitivity, inflammation, and energy metabolism (Villanueva-Carmona et al., 2023). In lean conditions, adipose tissue maintains an anti-inflammatory environment characterized by the presence of regulatory immune cells and the secretion of insulin-sensitizing adipokines such as adiponectin (Achari and Jain, 2017).

In contrast, obesity and chronic inflammatory states induce profound adipose tissue remodeling, marked by adipocyte hypertrophy, hypoxia, extracellular matrix expansion, and infiltration of pro-inflammatory immune cells, particularly M1-like macrophages (Kiepura et al., 2021). This shift results in increased production of pro-inflammatory cytokines (e.g., TNF-α, IL-6) and adipokines such as leptin and resistin, alongside reduced adiponectin levels (Huang K. et al., 2022). These changes promote systemic insulin resistance by impairing insulin signaling in skeletal muscle and liver, while also contributing to endothelial dysfunction and altered lipid metabolism (da Silva Rosa et al., 2020).

Adipose tissue inflammation is further amplified by lipolysis-driven release of free fatty acids, which act as signaling molecules that activate toll-like receptors and inflammasome pathways in distant organs (Miyamoto et al., 2017). Additionally, adipose-derived extracellular vesicles and lipid mediators contribute to the propagation of inflammatory signals, reinforcing the role of adipose tissue as a driver of systemic dysfunction (Bond et al., 2022).

Importantly, adipose tissue exhibits depot-specific differences, with visceral adipose tissue being more strongly associated with inflammatory and metabolic complications than subcutaneous depots (Nahmgoong et al., 2022). This heterogeneity adds another layer of complexity to inter-organ crosstalk, influencing disease risk and therapeutic responses (Nahmgoong et al., 2022).

From a pharmacological perspective, targeting adipose tissue inflammation and adipokine signaling represents a key strategy for restoring systemic metabolic balance (Xia et al., 2024; Westerink and Visseren, 2011). Interventions that promote adipose tissue remodeling toward an anti-inflammatory phenotype, including weight loss, pharmacotherapy, and modulation of immune cell function, are critical for improving inter-organ communication and reducing disease burden (Westerink and Visseren, 2011).

### 3.3 Hepatic integration of systemic signals

The liver functions as a central integrator of metabolic and inflammatory signals, coordinating systemic responses to nutrient availability, hormonal cues, and immune activation. Its strategic anatomical position and unique vascularization enable the liver to sense and respond to signals derived from the gut, adipose tissue, skeletal muscle, and circulating immune cells (Steinberg et al., 2025).

Hepatokines, including fetuin-A, fibroblast growth factor 21 (FGF21), angiopoietin-like proteins, and others, play pivotal roles in mediating hepatic communication with peripheral tissues (Szczepańska and Gietka-Czernel, 2022). Fetuin-A, for instance, promotes insulin resistance by inhibiting insulin receptor signaling and activating inflammatory pathways through toll-like receptor 4 (Thomas et al., 2020). In contrast, FGF21 exerts protective effects by enhancing glucose uptake, lipid oxidation, and energy expenditure, highlighting the dual nature of hepatokine signaling (Szczepańska and Gietka-Czernel, 2022).

During systemic inflammation, hepatic function is profoundly altered. Activation of inflammatory pathways such as NF-κB and JAK/STAT leads to the production of acute-phase proteins, increased gluconeogenesis, and dysregulated lipid metabolism (Ce et al., 2025). These changes contribute to hyperglycemia, dyslipidemia, and systemic inflammation, further propagating inter-organ dysfunction (Xu et al., 2025).

The liver also plays a critical role in immune regulation, acting as a site for immune cell activation and tolerance. Kupffer cells, the resident macrophages of the liver, respond to circulating inflammatory signals and contribute to cytokine production, thereby influencing systemic immune responses (Luo et al., 2018). Additionally, hepatic interactions with gut-derived signals *via* the portal circulation link liver function to the gut–liver axis, further integrating systemic signaling, defined in this review as the coordinated organism-wide physiological responses generated by the integration of endocrine, metabolic, inflammatory, and neural signals across multiple organs (Tilg et al., 2022).

Importantly, hepatic metabolic reprogramming during inflammation influences other organs through the release of metabolites, lipoproteins, and signaling molecules, reinforcing its role as a central node in inter-organ crosstalk (Sui and Chen, 2022). Dysregulation of these processes is a hallmark of metabolic diseases such as non-alcoholic fatty liver disease Metabolic Dysfunction-Associated Steatotic Liver Disease and type 2 diabetes mellitus (Fuentes-Barría et al., 2026; Cusi et al., 2025).

Building upon the central role of the liver as an integrative hub of metabolic and inflammatory signals, the muscle–adipose–liver axis represents a key network through which systemic homeostasis is regulated and, under pathological conditions, disrupted. Therapeutically, targeting hepatic signaling pathways offers significant potential for modulating systemic inflammation, as pharmacological agents that improve hepatic insulin sensitivity, reduce inflammation, and modulate hepatokine secretion can exert coordinated effects across multiple organs. However, given the bidirectional and multilayered nature of inter-organ communication, selective modulation of hepatic pathways without impairing essential metabolic functions remains a major challenge. In this context, Figure 2 provides an integrated overview of the dynamic crosstalk between skeletal muscle, adipose tissue, and the liver, highlighting the role of myokines, adipokines, hepatokines, extracellular vesicles, lipid mediators, and metabolites in shaping both physiological homeostasis and inflammation-driven dysfunction.

**FIGURE 2:**
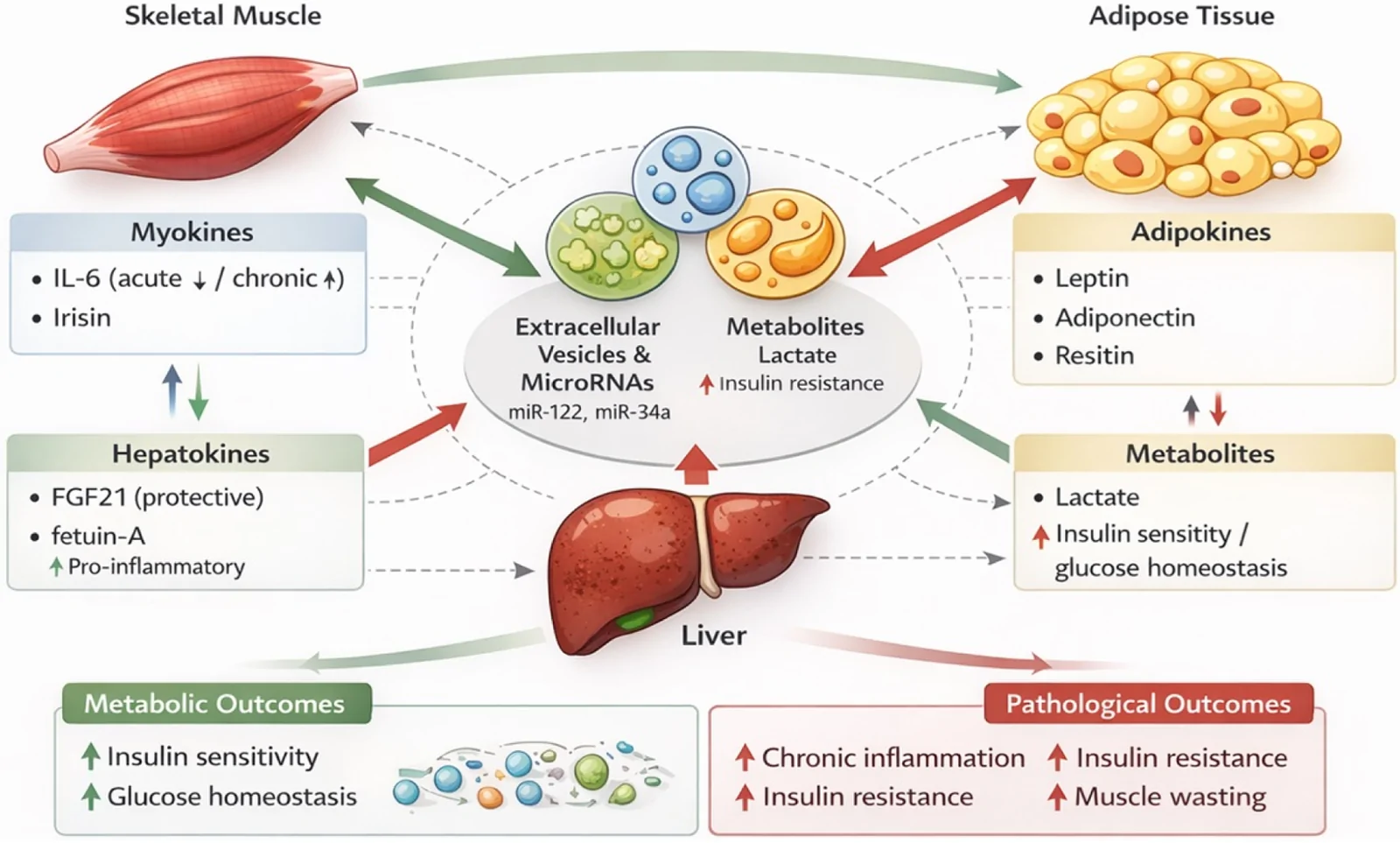
The muscle-adipose-liver axis in systemic inflammation. Source: own elaboration.

## 4 Pharmacological modulation of inter-organ crosstalk

The pharmacological modulation of inter-organ crosstalk represents an emerging paradigm in the treatment of systemic inflammatory and metabolic diseases (van Geffen et al., 2022). Rather than targeting isolated pathways or single organs, current strategies increasingly aim to reprogram interconnected signaling networks that integrate immune, metabolic, and endocrine responses (Tekampe et al., 2018; Parnham and Geisslinger, 2019). This shift reflects a growing recognition that therapeutic efficacy depends not only on local effects but also on the capacity to restore coordinated communication between organs (Li H. et al., 2025).

At the systems level, pharmacological interventions can influence inter-organ crosstalk by modulating cytokine signaling, extracellular vesicle dynamics, lipid mediator balance, and metabolic pathways (Parnham and Geisslinger, 2019). Importantly, many established drugs exert pleiotropic effects across multiple signaling layers, highlighting the need to reinterpret their mechanisms of action within a network-based framework. However, challenges remain in achieving specificity, minimizing off-target effects, and preserving physiological adaptive responses (Parnham and Geisslinger, 2019).

### 4.1 Targeting cytokine signaling pathways

Therapeutic targeting of cytokines has become a cornerstone of modern anti-inflammatory pharmacology, particularly through the use of monoclonal antibodies and receptor antagonists directed against key mediators such as IL-6, TNF-α, and IL-1β (Huang et al., 2024; Veerasubramanian et al., 2024). These interventions have demonstrated significant clinical efficacy across a wide spectrum of autoimmune and inflammatory diseases, including rheumatoid arthritis, inflammatory bowel disease, and cytokine storm syndromes, thereby validating cytokine signaling as a central driver of systemic pathology (Huang et al., 2024). Beyond their classical immunomodulatory roles, cytokines are now recognized as critical regulators of metabolic homeostasis and inter-organ communication, positioning them as key nodes within integrated immunometabolic networks (Liu et al., 2021).

At the mechanistic level, cytokine-targeted therapies disrupt central intracellular signaling hubs, including the JAK/STAT, NF-κB, and MAPK pathways, leading to broad transcriptional reprogramming that attenuates inflammatory cascades and downstream metabolic dysfunction (Pinzi et al., 2025). IL-6 receptor blockade, for example, not only reduces systemic inflammation but also modulates hepatic acute-phase protein synthesis, lipid metabolism, and glucose homeostasis, highlighting its pleiotropic effects across organ systems (Mo et al., 2024). Similarly, TNF-α inhibitors restore insulin signaling by reducing serine phosphorylation of insulin receptor substrates and decreasing activation of stress kinases such as JNK, while also improving endothelial function and vascular inflammation (Wu et al., 2023). Targeting IL-1β, particularly through inflammasome-related pathways, has further demonstrated benefits in reducing systemic inflammatory burden and cardiovascular risk, underscoring the clinical relevance of cytokine modulation beyond traditional autoimmune contexts (Huang et al., 2024; Dinarello, 2011).

Importantly, cytokine signaling operates within highly interconnected and dynamic networks characterized by redundancy, feedback loops, and context-dependent responses (Shvartsman et al., 2002). As such, the inhibition of a single cytokine pathway may lead to compensatory activation of parallel signaling routes, potentially limiting long-term therapeutic efficacy (Saxton et al., 2023). Moreover, cytokines exhibit dual and context-specific roles; for instance, IL-6 can exert both pro-inflammatory and anti-inflammatory effects depending on whether signaling occurs *via* membrane-bound or soluble receptors (Huang et al., 2024). This functional plasticity complicates therapeutic targeting, as indiscriminate inhibition may disrupt beneficial physiological processes, including host defense, tissue repair, and metabolic adaptation (Liu et al., 2021).

Despite their clinical success, cytokine-targeted therapies present important limitations. Broad immunosuppressive effects increase susceptibility to infections and may impair immune surveillance, raising concerns regarding long-term safety (Caprioli et al., 2012). Additionally, inter-individual variability in cytokine network architecture and receptor expression contributes to heterogeneous treatment responses, emphasizing the need for more personalized approaches (Huang et al., 2024; Pinti et al., 2023). The high cost and requirement for parenteral administration of many biologics further limit accessibility and long-term adherence (Lawrence et al., 2018).

### 4.2 Modulating extracellular vesicle pathways

Extracellular vesicle (EV)-based therapies represent a rapidly evolving and conceptually transformative field with significant potential for modulating inter-organ crosstalk in systemic inflammation and metabolic diseases (Mori et al., 2019). As key mediators of long-distance communication, EVs enable the transfer of complex molecular cargo—including proteins, lipids, metabolites, and regulatory RNAs—between tissues, thereby influencing cellular function in a highly coordinated and context-dependent manner (Mori et al., 2019). Pharmacological strategies targeting EV pathways can be broadly categorized into three main approaches: inhibition of EV biogenesis and release, blockade of EV uptake and biodistribution, and therapeutic exploitation of EVs as targeted delivery systems (Hao et al., 2021).

Inhibition of EV release has been explored as a strategy to limit the propagation of pathogenic signals across organs (Hao et al., 2021). This approach targets critical steps in EV biogenesis, including endosomal sorting and membrane budding processes (Hao et al., 2021). Pharmacological agents such as neutral sphingomyelinase inhibitors (e.g., GW4869) interfere with ceramide-dependent exosome formation, while modulators of ESCRT machinery and Rab GTPases disrupt vesicular trafficking and secretion (Liu et al., 2024). Additionally, cellular stress pathways, including NF-κB activation and hypoxia signaling, are known to regulate EV production, suggesting that upstream modulation of these pathways may indirectly influence EV release (Hao et al., 2021). By reducing EV secretion, these strategies aim to attenuate the dissemination of pro-inflammatory, pro-fibrotic, or oncogenic signals, particularly in conditions such as obesity, insulin resistance, cancer, and chronic inflammatory diseases (Hao et al., 2021).

Alternatively, blocking EV uptake by recipient cells represents a complementary strategy to disrupt inter-organ communication (Hao et al., 2021). EV internalization is mediated by a variety of mechanisms, including receptor-ligand interactions, endocytosis, phagocytosis, and membrane fusion, often regulated by surface molecules such as integrins, tetraspanins, and proteoglycans (Ghossoub et al., 2020). Pharmacological or molecular targeting of these surface interactions can reduce tissue-specific uptake of circulating EVs, thereby limiting their functional impact (Gyöngyösi et al., 2021). Importantly, the organotropism of EVs—determined in part by their surface protein composition—offers an opportunity to selectively modulate signaling pathways in specific tissues. However, the redundancy and diversity of EV uptake mechanisms present challenges for achieving precise and sustained inhibition (Nadeau et al., 2025).

Perhaps the most promising and rapidly advancing avenue is the use of engineered EVs as therapeutic carriers. Due to their intrinsic biocompatibility, low immunogenicity, and natural ability to cross biological barriers, EVs provide an attractive platform for targeted drug delivery (Song et al., 2022). Engineered EVs can be loaded with a wide range of therapeutic cargo, including small-molecule drugs, microRNAs, siRNAs, mRNAs, and gene-editing components such as CRISPR/Cas systems (Nobrega et al., 2025; Evers et al., 2022; Kumar M. A. et al., 2024). Surface modification strategies, including ligand conjugation or genetic engineering of donor cells, can further enhance tissue-specific targeting and functional delivery. For example, EVs derived from mesenchymal stem cells have demonstrated anti-inflammatory, immunomodulatory, and regenerative effects in multiple preclinical models, partly through the transfer of regulatory RNAs and bioactive proteins that modulate immune cell polarization and metabolic pathways (Zhang D. et al., 2024). Similarly, adipose- and muscle-derived EVs are being explored for their ability to restore physiological inter-organ communication in metabolic disorders (Jia et al., 2025).

Despite these promising developments, the clinical translation of EV-based therapies faces several significant challenges. Standardization of EV isolation, purification, and characterization remains a major limitation, as current methodologies yield heterogeneous populations with variable composition and function (Rai et al., 2021). Scalability and reproducibility are additional barriers, particularly for large-scale production under Good Manufacturing Practice (GMP) conditions. Cargo heterogeneity and incomplete understanding of cargo selection mechanisms further complicate therapeutic design, as unintended bioactive molecules may influence outcomes (Pachler et al., 2017). Regulatory considerations also pose challenges, given the hybrid nature of EVs as both biological and drug-like entities (Evers et al., 2022).

Moreover, a deeper understanding of EV biology is required to fully harness their therapeutic potential (Cheng and Hill, 2022; Gupta et al., 2021). Key areas of ongoing research include the mechanisms governing cargo sorting, the determinants of organ-specific targeting, and the dynamic regulation of EV release under physiological and pathological conditions (Fareez et al., 2022). Integration of EV research with multi-omics and systems biology approaches will be critical for identifying actionable nodes within EV-mediated communication networks (Meng et al., 2024).

Collectively, these strategies highlight the dual role of EVs as both drivers of disease propagation and promising therapeutic tools. Targeting EV pathways therefore represents a novel and versatile approach to modulating inter-organ crosstalk, with the potential to achieve highly specific and context-dependent therapeutic effects in complex systemic diseases (Meng et al., 2024; Roudi et al., 2023).

### 4.3 Lipid mediator-based therapies

Pharmacological targeting of lipid mediator pathways has long been a cornerstone of anti-inflammatory therapy, with non-steroidal anti-inflammatory drugs (NSAIDs) representing one of the most widely used and clinically established classes (Arfeen et al., 2024). These agents primarily inhibit COX enzymes—COX-1 and COX-2—thereby reducing the synthesis of prostaglandins and thromboxanes, which are key mediators of inflammation, pain, and fever (Vardhini et al., 2022). The widespread use of NSAIDs underscores the central role of eicosanoid signaling in inflammatory processes; however, their non-selective mechanism of action and associated adverse effects, including gastrointestinal toxicity, renal dysfunction, and cardiovascular risk, highlight important limitations in their long-term use (Arfeen et al., 2024).

While effective in suppressing acute inflammatory responses, traditional NSAIDs predominantly target pro-inflammatory pathways without addressing the active resolution phase of inflammation (Arfeen et al., 2024). Resolution is now recognized as a highly regulated and biologically active process mediated by specialized pro-resolving lipid mediators (SPMs), including resolvins, protectins, and maresins, which are biosynthesized from omega-3 polyunsaturated fatty acids (Berrocal-Navarrete et al., 2026). These mediators orchestrate the termination of inflammation by promoting macrophage-mediated clearance of cellular debris, limiting neutrophil infiltration, and restoring tissue homeostasis (Pervizaj-Oruqaj et al., 2024). The inability of conventional anti-inflammatory drugs to engage these pro-resolving pathways has driven the development of novel therapeutic strategies aimed at rebalancing the lipid mediator network rather than simply suppressing inflammatory signals (Bernela et al., 2023).

Selective modulation of lipid mediator receptors represents a more refined pharmacological approach with the potential to improve efficacy and safety. Targeting specific prostaglandin receptors (e.g., EP receptors) or leukotriene receptors (e.g., BLT and CysLT receptors) allow pathway-specific intervention, reducing off-target effects associated with global enzyme inhibition (Alhallak et al., 2024; Zhao et al., 2020; Luginina et al., 2023). In parallel, the development of synthetic analogs of SPMs and agonists of their receptors has opened new avenues for promoting inflammation resolution (Zhao et al., 2021; Byrne and Guiry, 2024). These agents have demonstrated promising results in preclinical models, enhancing tissue repair, reducing chronic inflammation, and preserving host defense mechanisms—an important advantage over broadly immunosuppressive therapies (Ji, 2023).

Beyond their local effects, lipid mediators play a critical role in coordinating inter-organ communication, positioning them as central regulators within systemic immunometabolic networks (Batista-Gonzalez et al., 2020). Eicosanoid signaling pathways interact extensively with cytokine cascades and metabolic processes, forming integrated regulatory circuits that influence both immune and metabolic homeostasis (Kang and Kishimoto, 2021). For instance, adipose tissue-derived lipid mediators can modulate hepatic gluconeogenesis, lipid metabolism, and insulin sensitivity, while hepatic eicosanoids can regulate systemic inflammatory tone and vascular function (Kang and Kishimoto, 2021). Additionally, lipid mediators influence skeletal muscle metabolism and immune cell activation, further reinforcing their role in the muscle–adipose–liver axis (Shimizu et al., 2015). This system’s level integration highlights the potential of lipid-targeted therapies to exert coordinated effects across multiple organs (Schneider Alves et al., 2022).

Recent advances in lipidomics have significantly enhanced the ability to profile lipid mediator networks with high resolution, enabling the identification of disease-specific lipid signatures and dynamic changes in pro-inflammatory *versus* pro-resolving mediators (Arita, 2012). This has facilitated a shift toward precision medicine approaches, where therapeutic strategies are tailored based on individual lipidomic profiles and network dynamics (Zandl-Lang et al., 2023). Systems pharmacology further complements this approach by integrating lipid mediator pathways with broader biological networks, allowing the prediction of drug effects and identification of optimal intervention points (Chen et al., 2022).

Future therapeutic strategies are therefore likely to focus on the precision modulation of lipid mediator networks, aiming to restore the balance between inflammation initiation and resolution. This includes not only the development of receptor-selective drugs and SPM-based therapies but also combination approaches that simultaneously target multiple nodes within the lipid signaling network. Such strategies hold promises for achieving more effective, context-dependent, and individualized treatments for systemic inflammatory and metabolic diseases, while minimizing adverse effects associated with conventional anti-inflammatory therapies.

### 4.4 Metabolic and immunometabolic interventions

Targeting metabolic pathways has emerged as a powerful strategy for modulating systemic inflammation and inter-organ crosstalk (Cibrian et al., 2020). Drugs traditionally used for metabolic diseases are now recognized to exert broad immunomodulatory and anti-inflammatory effects, reflecting the deep integration between metabolism and immune function (Cibrian et al., 2020; DeLeon et al., 2025).

Metformin, one of the most widely used antidiabetic agents, exemplifies this concept. Through activation of AMPK and modulation of mitochondrial function, metformin influences glucose metabolism, reduces hepatic gluconeogenesis, and attenuates inflammatory signaling pathways such as NF-κB. These effects extend to multiple organs, improving systemic metabolic and inflammatory profiles (González-Casanova et al., 2025).

Similarly, glucagon-like peptide-1 (GLP-1) receptor agonists exert pleiotropic effects that include enhanced insulin secretion, reduced appetite, and modulation of inflammatory pathways in adipose tissue, liver, and cardiovascular system (Kahles et al., 2026). Sodium–glucose cotransporter 2 (SGLT2) inhibitors further contribute to systemic regulation by promoting glycosuria, improving metabolic efficiency, and reducing inflammation and oxidative stress (Winiarska et al., 2021). More recently, imeglimin, a first-in-class tetrahydrotriazine, has emerged as a promising metabolic therapy through its ability to improve mitochondrial bioenergetics, reduce oxidative stress, and enhance insulin sensitivity (Fukunaga et al., 2025; Uto et al., 2024). Beyond glycemic control, preclinical and emerging clinical evidence suggests that imeglimin may attenuate hepatic steatosis, inflammation, and fibrosis, supporting its potential role in the management of metabolic dysfunction-associated fatty liver disease (MAFLD) by simultaneously targeting liver metabolism and systemic metabolic homeostasis (Fukunaga et al., 2025; Uto et al., 2024).

Beyond these established therapies, emerging approaches aim to directly target immunometabolic pathways, including modulation of mitochondrial metabolism, redox balance, and key metabolic intermediates. In parallel, ferroptosis, an iron-dependent form of regulated cell death characterized by lipid peroxidation, has emerged as an important mechanism linking systemic inflammation with multi-organ dysfunction (Tang et al., 2021). Dysregulated ferroptosis contributes to tissue injury in the liver, heart, skeletal muscle, kidney, pancreas, and adipose tissue, thereby disrupting inter-organ communication through the release of inflammatory mediators, damage-associated molecular patterns, and extracellular vesicles (Tang et al., 2021; Liang et al., 2022) Consequently, therapeutic strategies targeting ferroptosis may simultaneously preserve organ function and restore systemic metabolic homeostasis. These strategies seek to reprogram immune cell function and restore metabolic homeostasis across organs (Xu R. et al., 2024).

Importantly, metabolic interventions influence not only intracellular pathways but also circulating mediators, including metabolites, cytokines, and EVs, thereby reshaping inter-organ communication networks (Limpitikul et al., 2025). Recent advances in multi-omics technologies—including genomics, transcriptomics, epigenomics, proteomics, metabolomics, lipidomics, and extracellular vesicle omics—have substantially improved the characterization of these inter-organ communication networks. The integration of multi-omics datasets with systems biology and artificial intelligence approaches enables the identification of molecular signatures, signaling pathways, and therapeutic targets underlying systemic inflammatory diseases, thereby facilitating precision medicine and personalized therapeutic strategies. This systems-level impact positions immunometabolic therapies as a promising avenue for the treatment of complex diseases characterized by systemic inflammation (Pålsson-McDermott and O'Neill, 2020).

Building upon the concept of network-based pharmacological modulation, it is increasingly evident that therapeutic interventions extend beyond isolated intracellular targets to influence systemic communication pathways. Importantly, metabolic and immunometabolic therapies modulate not only intracellular signaling cascades but also circulating mediators, including metabolites, cytokines, and extracellular vesicles, thereby reshaping inter-organ communication networks at multiple levels. This systems-level perspective highlights how pharmacological agents can simultaneously impact diverse tissues within the muscle–adipose–liver axis, ultimately restoring metabolic and inflammatory homeostasis. In this context, Table 2 summarizes representative drug classes, their primary molecular targets, and their systemic effects across organs, emphasizing their translational and clinical relevance in the management of systemic inflammatory and metabolic diseases.

**TABLE 2:** Representative drugs and multi-organ effects.

Drug/Class	Primary target	Secondary effects (Cross-talk)	Organ impact	Clinical relevance
Metformin	AMPK	↓ NF-κB, ↓ cytokines	Liver, muscle	T2DM and anti-inflammatory
GLP-1 RAs	GLP-1R	↓ appetite, ↓ inflammation	Adipose, liver	Obesity and T2DM
SGLT2 inhibitors	SGLT2	↓ oxidative stress	Kidney, liver	CV protection
Anti-IL-6	IL-6R	↓ acute-phase response	Immune cells, liver	Autoimmune diseases

Source: own elaboration.

Complementarily, Figure 3 provides an integrated overview of the main pharmacological strategies and their points of action within inter-organ crosstalk networks, illustrating their multi-target and network-based effects.

**FIGURE 3:**
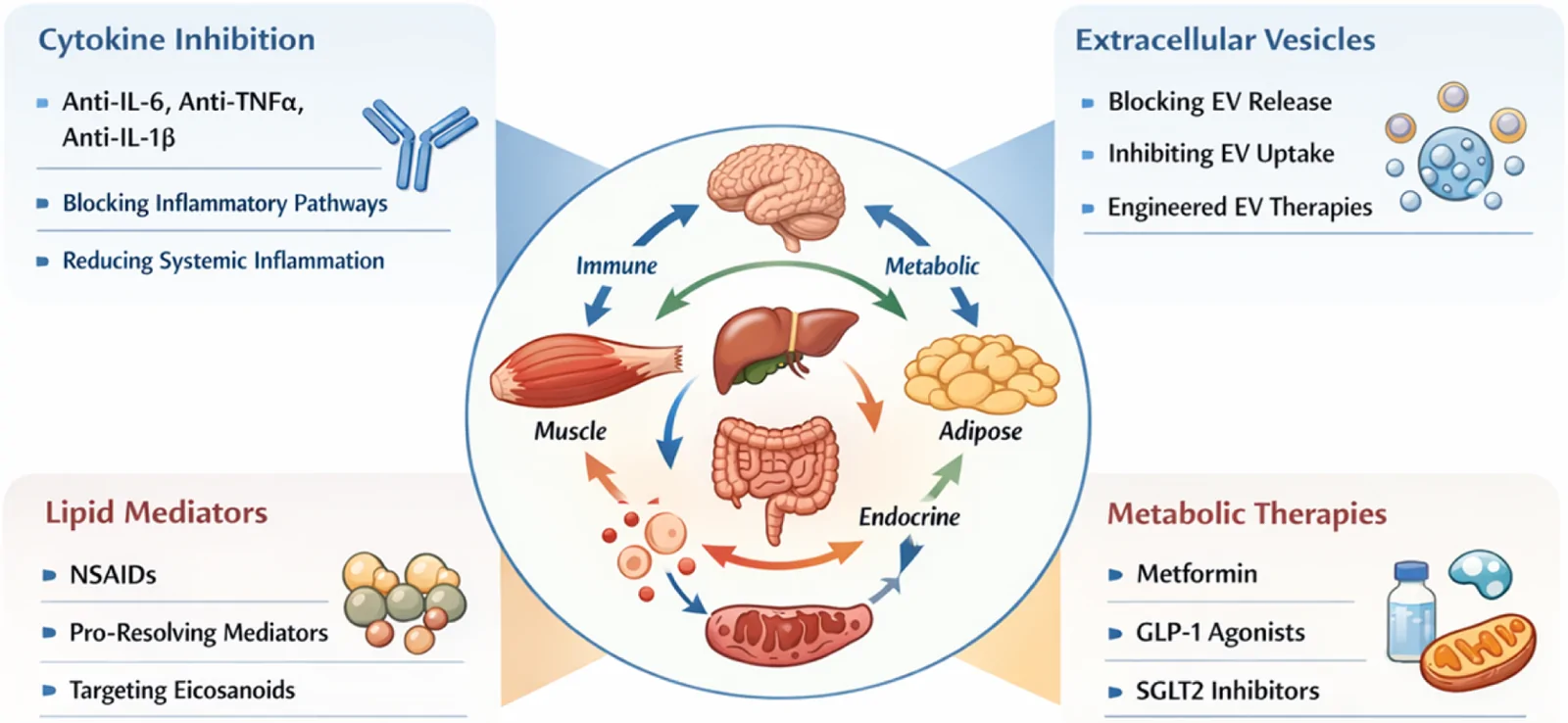
Pharmacological modulation of inter-organ crosstalk. Source: own elaboration.

## 5 Multi-omics and systems pharmacology approaches

The increasing recognition of inter-organ crosstalk as a central determinant of systemic inflammation has driven the adoption of multi-omics and systems pharmacology approaches to unravel the complexity of these networks (Xiao et al., 2025). Traditional reductionist methodologies, while instrumental in identifying individual pathways, are insufficient to capture the dynamic and multilayered interactions that define inter-organ communication (Liu et al., 2025). In this context, integrative omics technologies provide an unprecedented opportunity to map the molecular architecture of systemic inflammation at a systems level (Li L. et al., 2025).

Multi-omics platforms—including transcriptomics, proteomics, metabolomics, lipidomics, and EV profiling—enable the simultaneous characterization of multiple layers of biological regulation (Jiang et al., 2023). Transcriptomics provides insights into gene expression programs across tissues, while proteomics captures post-translational modifications and signaling pathway activation (Cui et al., 2025). Metabolomics and lipidomics further extend this analysis by identifying bioactive metabolites and lipid mediators that function as signaling entities in inter-organ communication (Wang et al., 2020). Importantly, EV-associated omics adds an additional dimension by linking molecular cargo to specific tissue origins and intercellular transfer mechanisms (Wang J. et al., 2026).

A critical advancement in this field is the integration of these datasets into multi-layered networks, allowing the identification of key regulatory nodes and signaling hubs that govern systemic responses (Katoh, 2018). Network-based analyses, including co-expression networks, causal inference modeling, and machine learning algorithms, facilitate the reconstruction of inter-organ communication pathways and the identification of biomarkers associated with disease progression or therapeutic response (Kumar R. et al., 2024).

Systems pharmacology extends these concepts by integrating pharmacokinetics, pharmacodynamics, and network biology to predict drug effects at the organism level (van Hasselt and Iyengar, 2019). Rather than focusing on single targets, systems pharmacology considers drugs as modulators of complex biological networks, enabling the identification of off-target effects, synergistic interactions, and context-dependent responses (Li et al., 2023). This approach is particularly relevant for drugs with pleiotropic actions, such as metabolic and anti-inflammatory agents, whose systemic effects cannot be fully explained by single-pathway models.

In the context of inter-organ crosstalk, systems pharmacology enables the identification of mechanistic nodes amenable to therapeutic intervention, such as key cytokine pathways, metabolic checkpoints, or EV-mediated signaling routes. Furthermore, the integration of patient-derived multi-omics data supports the development of precision medicine strategies, allowing for the stratification of individuals based on molecular profiles and predicted therapeutic responses.

Despite these advances, several challenges remain, including data heterogeneity, limited standardization across platforms, and the need for robust computational frameworks capable of integrating high-dimensional datasets. Future progress will depend on the development of interoperable data infrastructures, improved bioinformatic tools, and closer integration between experimental and computational approaches. Ultimately, multi-omics and systems pharmacology hold the potential to transform our understanding of systemic inflammation and to guide the development of more effective, personalized therapies targeting inter-organ communication networks.

To address the inherent complexity of inter-organ crosstalk in systemic inflammation, multi-omics and systems pharmacology approaches have emerged as powerful integrative frameworks that move beyond traditional reductionist paradigms. By simultaneously capturing multiple layers of biological regulation—including transcriptomics, proteomics, metabolomics, lipidomics, and extracellular vesicle profiling—these approaches enable a comprehensive characterization of the molecular networks underlying inter-organ communication. The integration of high-dimensional datasets through advanced computational methods, such as network analysis and machine learning, facilitates the identification of key regulatory nodes, signaling hubs, and potential biomarkers associated with disease progression and therapeutic response. In parallel, systems pharmacology extends this integrative perspective by linking molecular networks with pharmacokinetic and pharmacodynamic properties, allowing the prediction of drug effects at the organism level. As illustrated in Figure 4, this multi-layered framework connects omics data acquisition with network-based analysis, biological insight generation, and clinical application, ultimately supporting the development of precision medicine strategies. Despite these advances, challenges such as data heterogeneity, limited standardization, and translational gaps remain critical barriers, underscoring the need for continued methodological and computational innovation.

**FIGURE 4:**
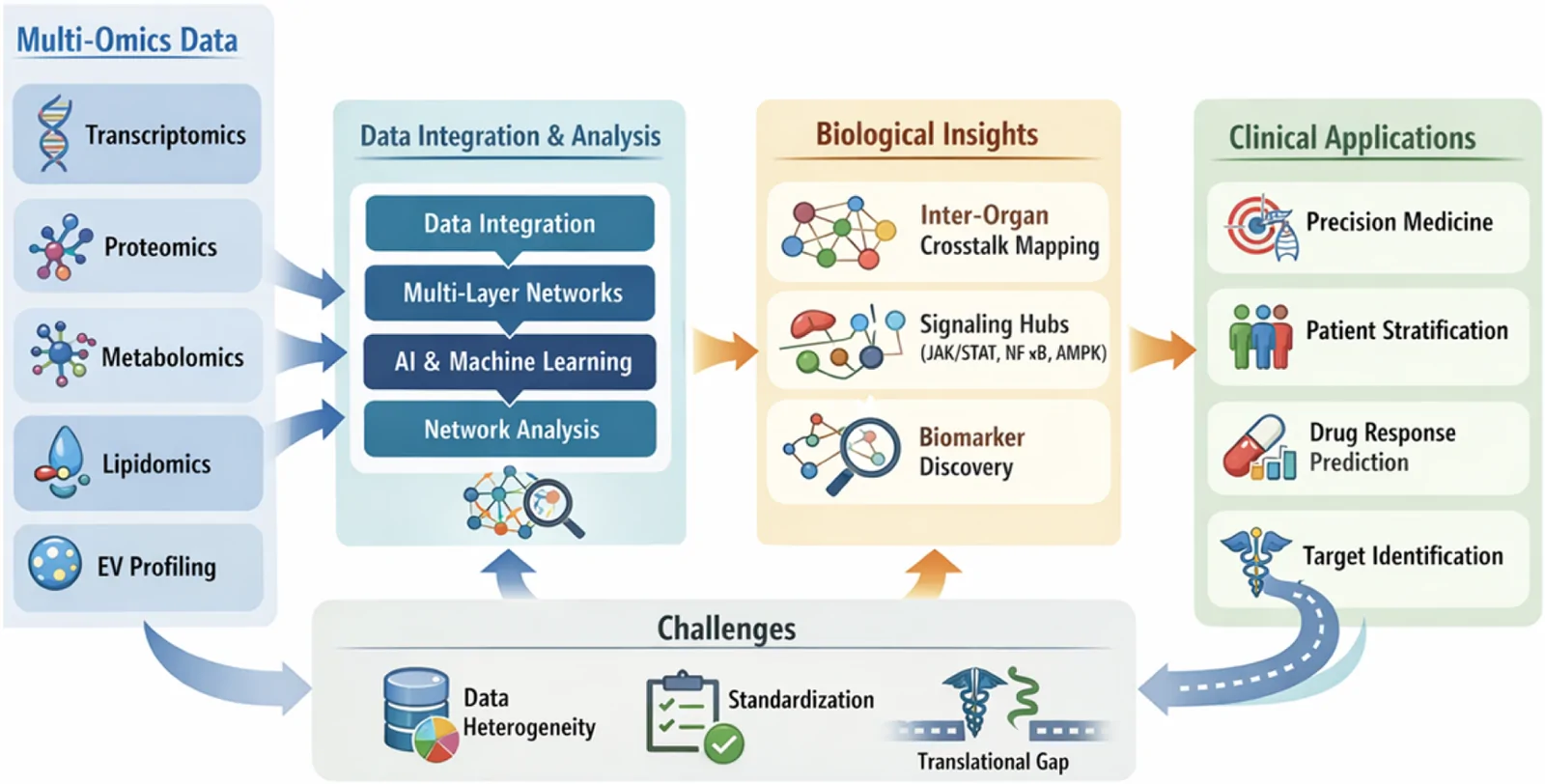
Multi-omics systems pharmacology framework. Source: own elaboration.

## 6 Challenges and future directions

Despite substantial advances in the understanding of inter-organ crosstalk during systemic inflammation, significant challenges remain that limit the translation of mechanistic insights into clinical applications. One of the primary obstacles is the intrinsic complexity and dynamic nature of inter-organ communication networks, which involve multiple layers of regulation, including cytokines, metabolites, lipid mediators, and extracellular vesicles. These networks are highly context-dependent, varying across physiological states, disease conditions, and temporal scales, making it difficult to define universal mechanisms or therapeutic targets.

Inter-individual variability represents another major challenge. Genetic background, age, sex, microbiome composition, and environmental factors all influence the structure and function of inter-organ communication networks. This heterogeneity complicates the identification of consistent biomarkers and limits the generalizability of experimental findings. As a result, there is a growing need for stratified and personalized approaches that account for patient-specific characteristics and molecular profiles.

Experimental limitations further constrain progress in this field. Many current models, including *in vitro* systems and animal models, fail to fully recapitulate the complexity of human inter-organ crosstalk. While advances such as organ-on-chip technologies and multi-organ culture systems offer promising alternatives, their scalability and physiological relevance remain under active development. Bridging the gap between experimental models and human physiology is essential for improving translational outcomes.

From a methodological perspective, the integration of multi-omics data poses significant challenges related to data standardization, reproducibility, and computational analysis. The lack of unified frameworks for data integration and interpretation can lead to inconsistent results and hinder the identification of robust mechanistic insights. Addressing these issues will require the development of standardized protocols, shared databases, and advanced analytical tools capable of handling high-dimensional and multi-scale data.

Looking forward, several key directions are likely to shape the future of this field. First, the integration of multi-scale data, spanning molecular, cellular, tissue, and organismal levels, will be critical for constructing comprehensive models of inter-organ communication. Second, advances in artificial intelligence and machine learning will enhance the ability to identify patterns, predict disease trajectories, and optimize therapeutic strategies. Third, the identification of clinically relevant biomarkers, particularly those derived from circulating mediators such as EVs and metabolites, will be essential for early diagnosis, patient stratification, and treatment monitoring.

Finally, the development of targeted and network-based therapeutic strategies represents a promising avenue for improving clinical outcomes. Rather than focusing on single pathways, future interventions are likely to aim at restoring the balance of inter-organ communication networks, leveraging combination therapies and context-specific modulation. Achieving this goal will require close collaboration between basic scientists, clinicians, and computational researchers, as well as a shift toward integrative and systems-level thinking in both research and clinical practice.

## 7 Conclusion

Systemic inflammation is a network-driven process sustained by dynamic inter-organ communication, with the muscle–adipose–liver axis acting as a central regulatory hub. Its dysregulation contributes to the development of metabolic and inflammatory diseases.

Inter-organ crosstalk is mediated by interconnected signaling layers, including cytokines, extracellular vesicles, lipid mediators, and metabolites, which collectively determine systemic outcomes. Pharmacological strategies targeting these pathways show promise, particularly as emerging approaches aim for more precise and context-specific modulation.

Advances in multi-omics and systems pharmacology are facilitating the identification of key regulatory nodes and biomarkers, supporting precision medicine. Targeting inter-organ communication thus represents a critical step toward more integrative therapeutic strategies in systemic inflammation.
